# The Effects of Transdermal Nicotine Patches on the Cardiorespiratory and Lactate Responses During Exercise from Light to Moderate Intensity: Implications for Exercise Prescription during Smoking Cessation

**DOI:** 10.3390/medicina55070348

**Published:** 2019-07-07

**Authors:** Takashi Nakagata, Kosuke Fukao, Hiroyuki Kobayashi, Shizuo Katamoto, Hisashi Naito

**Affiliations:** 1 Graduate School of Health and Sports Science, Juntendo University, Hiraka-gakuendai 1-1, Inzai, Chiba 270-1695, Japan; 2 National Institute of Health and Nutrition, National Institutes of Biomedical Innovation, Health and Nutrition, 1-23-1 Toyama, Shinjuku-ku, Tokyo 162-8636, Japan; 3 Mito Medical Center, Tsukuba University Hospital, 1-1-1 Tennodai, Tsukuba, Ibaraki 310-0015, Japan

**Keywords:** smoking habits, smoking cessation, cardiovascular disease, mortality, weight gain, exercise prescription, exercise program, heart rate, clinical setting

## Abstract

*Background and objectives:* Exercise can help ease withdrawal symptoms of smokers. However, there is little information about the physiological responses, such as cardiorespiratory and lactate (La) responses, during exercise from light to moderate intensity combined with transdermal nicotine patches (TNPs) in smokers. This study aimed to investigate the effect of TNPs on the cardiorespiratory and La responses during exercise at light to moderate intensity. *Materials and Methods:* Fourteen young men (8 non-smokers, 6 current smokers) aged 20 to 26 years participated in this study. They performed an incremental graded submaximal exercise test using an electromagnetic cycle ergometer set from 30 to 210 W with (TNP condition) or without a TNP (control condition) in a random order. The TNP was applied to the left arm 8–10 h prior to starting the exercise to achieve the peak level of blood nicotine concentration. Heart rate (HR), rate of perceived exertion (RPE), oxygen consumption (*V*O_2_), ventilation (*V*E), and blood La at rest and during exercise were measured and analyzed. *Results:* The HR at rest was significantly higher in the TNP condition than in the control condition (TNP; 74.7 ± 13.8 bpm, control; 65.3 ± 10.8 bpm, *p* < 0.001). There was no interaction (condition × exercise intensity) between any of the variables, and *V*O_2_, *V*E, RPE, and La during exercise were not significantly different between the conditions. However, HR during exercise was 6.7 bpm higher on average in the TNP condition. *Conclusions:* The HR during exercise was greater at light to moderate intensity with a TNP. Our study results will guide clinicians or health professionals when prescribing exercise programs combined with TNPs for healthy young smokers.

## 1. Introduction

Smoking is a major cause of cancer, cardiovascular diseases, diabetes, and many other diseases; however, smoking cessation reduces the risk of developing these major chronic diseases and extends life expectancy [1,2,3]. The World Health Organization has set a goal of reducing the global smoking rate by 30% by 2025 [4]. The provision of a smoking cessation program for smokers is essential for public health [5].

However, smoking cessation is not necessarily easy for smokers owing to different factors. First, nicotine withdrawal symptoms, such as cravings, anxiety and irritability, depression, and smoking urges, appear in the early stages of smoking cessation [6]. For this reason, the majority of smokers who attempt to quit smoking relapsed. To solve this problem and reduce nicotine withdrawal symptoms, nicotine replacement therapy (NRT), such as the transdermal nicotine patches (TNPs) and nicotine gum, in addition to counseling are the most common methods for smokers in the clinical setting. In particular, TNPs are easy to use and provide a slow, steady level of nicotine over a long period as compared to nicotine gum [7]. Furthermore, TNPs can be applied safely in normal individuals [8,9] and various populations (e.g., patients with cardiac disease, pregnant women) [10,11] using low or high nicotine doses.

Physical activity and exercise have been proposed as an aid for smoking cessation [12], and performing physical activity and exercise may have the potential to lessen some of the negative consequences of smoking withdrawal [13,14]. For example, acute bouts of exercise (e.g., walking, cycle ergometer) have been found to have a positive effect in the reduction of nicotine withdrawal symptoms and smoking craving [15,16,17], which are important factors that could lead to smoking relapse. In addition, a combination of exercise and NRT is effective for smoking cessation [18]. Thus, it is recommended to perform physical activity and exercise during smoking cessation to attenuate the nicotine withdrawal symptoms and smoking craving.

However, nicotine affects the human physiological responses, such as cardiovascular responses. For example, heart rate (HR) is controlled by the sympathetic and parasympathetic nervous systems, and the parasympathetic nervous system predominates as compared to the sympathetic nervous system at the resting state [19,20], although nicotine stimulates the central, sympathetic, and parasympathetic nervous systems as well as the secretion of catecholamine (adrenaline and noradrenaline) [21]. As a result, TNPs increase the HR by 6–10 bpm [22,23]. In addition, previous studies investigating the physiological responses during physical activity and exercise combined with nicotine products reported that HR during exercise was higher in nicotine conditions than in controls [24,25,26]. In contrast, Mundel et al. using TNPs reported that there were no significant differences in cardiorespiratory responses (HR, ventilation (*V*E), and oxygen consumption (*V*O_2_)) between TNP and control experiments. However, they investigated the cardiorespiratory responses during exercise with TNPs at only one exercise intensity until exhaustion (77% maximum *V*O_2_), and no study has been conducted to investigate the effect of TNPs on the cardiorespiratory responses during exercise at lower intensities. As mentioned previously, nicotine stimulates the sympathetic nervous systems and the secretion of catecholamine; thus, we hypothesize that TNPs increase cardiorespiratory responses during lower exercise intensity as compared to exercises without TNPs. In addition, exercise intensity should be dependent on age, fitness level, and health condition, and it is common to perform exercises at light to moderate intensity, such as walking and cycle ergometer exercise, at the beginning of the exercise program in a clinical setting. Therefore, a further study is needed to examine cardiorespiratory responses during exercise at light to moderate intensity for smoking cessation combined with TNPs.

The purpose of this study was to investigate the effect of TNPs on the cardiorespiratory and lactate (La) responses during exercise at light to moderate intensity.

## 2. Materials and Methods

### 2.1. Participants

This study included 14 adult male individuals (8 non-smokers, 6 current smokers) aged 20 to 26 years (age, 23.3 ± 2.3 years; height, 174.3 ± 5.6 cm; weight, 67.6 ± 10.6 kg; body mass index (BMI), 22.2 ± 2.5 kg/m^2^). They were recruited from our university through printed advertisements and by word of mouth. Participants underwent an annual health examination at their university and were found to have normal blood pressure (BP) and electrocardiograms and had no history of established cardiovascular disease, pulmonary disease, or neurological disease. We excluded participants taking medicines or supplements. Table 1 shows the participant characteristics and anthropometric data. All participants had regular exercise habits (1–3 days per week). Prior to the study, all included individuals provided written consent to participate after receiving information about the procedures and purpose of the study. The study protocol was approved by the Research Ethics Review Board of the Juntendo University Graduate School of Health and Sports Science (approval no. 27–24).

The sample size was calculated with GPower 3.1.3 (Dusseldorf, Germany) based on an assumption of the clinically significant differences in HR responses between TNPs and without TNPs apriori as effect size d = 0.9 of two dependent means (matched pairs), with α error prob = 0.05 and power (1–β) = 0.8. The required total sample size was estimated to be *n* = 13.

### 2.2. Experimental Design

The study was a random crossover single-blind design (with or without TNP conditions on two separate days). In session 1, the participants performed either the TNP or control sessions. In session 2, they performed either the TNP or control sessions, all the participants completed both the experiments on two separate days within one week. To eliminate the influence of execution order, the allocation of the TNP or control to sessions 1 and 2 was randomized among participants. The participants in this study refrained from doing any strenuous physical activity, including general exercise, from the day prior to the start of the experiment, and they started fasting (no water restriction) four hours before starting the experiment. Height and body weight were measured before the exercise. For each participant, the resting oxygen consumption (*V*O_2_) was measured in both sessions using an indirect calorimeter (AE-300s, Minato Medical Science Co., Ltd., Osaka, Japan) while sitting on a cycle ergometer and maintaining a resting position for 5 min with a face mask attached. After measuring the resting *V*O_2_, each participant performed session 1 or 2. All the measurements were carried out in a laboratory, where the temperature and humidity of the internal atmosphere were adjusted to 20 °C and 50%, respectively.

### 2.3. Transdermal Nicotine Patch (TNP)

The present study used TNPs (Novartis, 10 cm^2^, 17.5 mg nicotine patch, release rate 7 mg nicotine per 24 h). A TNP was applied to the left arm at 21:00 to 22:00 to ensure that the blood nicotine concentration reached the maximum value during exercise, because previous studies have reported that the blood nicotine concentration reached the maximum level at 8–10 h after TNP application [7,27]. In the control condition, a seal with similar size but without nicotine was attached to the left arm at 21:00 to 22:00. Furthermore, we attached another seal onto the TNPs and the control seals so as to blind the participants to the experimental protocol. Given that TNPs may cause headache and nausea and those could affect the study results, all participants underwent TNP application with a lower nicotine content for 10 h prior to the experiment, as a pre-screening method; only those who did not experience adverse effects, such as headaches and nausea, were included.

### 2.4. Incremental Graded Exercise Test and Indirect Calorimetry Measurement

All participants performed an incremental graded submaximal exercise test using an electromagnetic cycle ergometer (PowerMax Vll, COMBI WELNESS. Tokyo, Japan). The graded exercise test consisted of eight incremental stages, starting at 0.5 kp for 4 min, and then increasing to 0.5 kp (30 W) every 4 min until 3.5 kp (210 W). Participants were asked to maintain the cadence at 60 rpm and to adjust the rhythm with the sound of a metronome during the exercise test. Respiratory gas measurement using indirect calorimetry (AE-300s, Minato Medical Science Company, Ltd.) and a face mask was carried out in our laboratory as previously described [28]. All data were processed every 30 s, and the *V*O_2_ and carbon dioxide production (*V*CO_2_) were measured. The last 2 min in each stage was used to evaluate the *V*O_2_ and *V*CO_2_.

### 2.5. Heart Rate, Ratings of Perceived Exertion, and Blood Lactate Concentration

HR was recorded during the whole experiment using an electrocardiogram device (Fukuda Electronics Co., Ltd. Tokyo, Japan). Three beats were recorded 15 s before the end of each stage; the average value was taken as the HR of each stage. The rate of perceived exertion (RPE) was recorded using the 6–20-step Borg scale [29] after each stage. Blood samples (20 μL) were collected from the earlobe using a capillary tube [30] using a Biosen S-Line device (EKF Diagnostik, Barleban, Germany) before the exercise and immediately after each stage in both experiments. The La threshold (LT) is the initial breakpoint in the elevation of blood La concentrations. The determination of LT was based on visual inspection by five trained staff members, and the mean LT was calculated from three of the five results [31].

### 2.6. Statistical Analysis

Microsoft Office Excel 2017 and PASW Statistics version 20.0 (SPSS, IBM Inc. BM Corp., Armonk, NY, USA) were used for data processing and statistical analyses, respectively. All the variable results are presented as mean ± standard deviation. To examine the main effect (Condition and Exercise intensity) and interaction (condition × exercise intensity), two-way repeated analysis of variance (ANOVA) was conducted for each variable. One-way repeated ANOVA was conducted to examine the main effect for each variable in each condition separately if a significant interaction was observed. A paired t-test was conducted to determine the significant differences between TNP and control experiments. The statistical significance level was set at 0.05.

## 3. Results

All participants successfully completed both experimental sessions.

**Figure 1:**
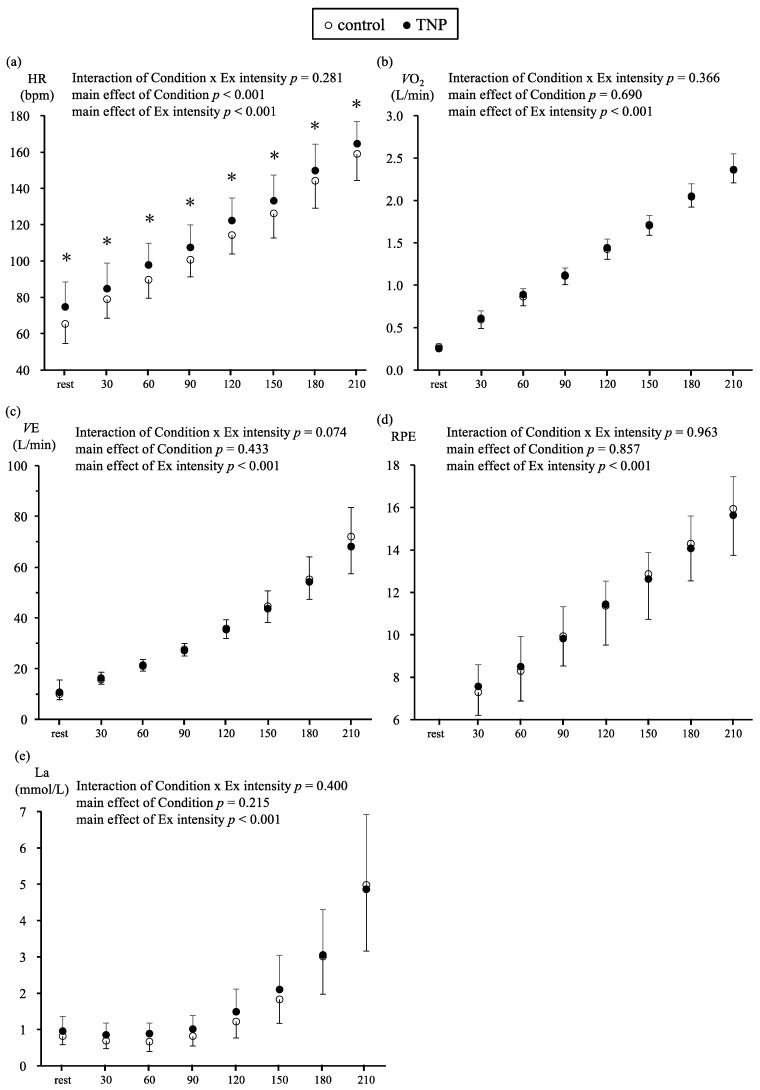
Physiological responses at rest and during exercise (Ex) for the transdermal nicotine patch (TNP) and control conditions. (**a**) heart rate (HR), (**b**) oxygen consumption (*V*O_2_), (**c**) ventilation (*V*E), (**d**) rate of perceived exertion (RPE) and (**e**) lactate (La). The symbol “white circle” is the control condition, and the symbol “black circle” is the TNP condition. Two-way repeated analysis of variance (ANOVA) shows no significant interaction between condition and exercise intensity, and heart rate (HR) during exercise is significantly higher in the TNP condition than in the control condition (*p* < 0.001). * *p* < 0.05, significant difference between conditions.

Figure 1 shows the physiological responses at rest and during the exercise test in both experimental sessions. HR at rest was significantly higher in the TNP condition than in the control condition (TNP; 74.7 ± 13.8 bpm, control; 65.3 ± 10.8, *p* < 0.001). Other variables at rest, *V*O_2_, *V*E, and La, were not significantly different between the two conditions.

During the exercise test, two-way repeated ANOVA shows no interaction (condition × exercise intensity) for all variables, and *V*O_2_, *V*E, La, and RPE during exercise were not significantly different between the TNP and control conditions. However, HR was significantly higher in the TNP condition than in the control condition (*p* < 0.001), HR during exercise was 6.7 bpm higher on average in the TNP condition than in the control condition. LT was not significantly different (TNP; 117.8 ± 18.6 W, control; 116.6 ± 17.2 W, *p* = 0.518) between the two conditions.

## 4. Discussion

It has been reported that exercise suppresses nicotine withdrawal symptoms and smoking cessation at the same time as NRT, and now combining NRT and exercise is expected to play an important role in quitting smoking [18]. However, the physiological responses, such as cardiorespiratory and La responses, during exercise when using TNPs have not been extensively studied. It is important to investigate these physiological responses because nicotine increases resting HR and BP. In this study, we investigated these responses during submaximal exercise at light to moderate intensity with or without TNPs. The main finding of the study is that TNPs increased HR by 9–10 bpm at rest and during exercise (6.7 bpm higher on average in the TNP condition). However, the relationship between exercise intensity and HR responses during exercise was not significantly different between both conditions, and TNP did not increase the HR throughout the incremental exercise. In addition, there were no significant differences in *V*O_2_, *V*E, RPE, and La concentration.

The parasympathetic nervous system predominates and controls the HR at the resting state [19,20]. However, nicotine increases HR owing to different factors, such as stimulation of the central nervous system, vasodilation, and stimulation of catecholamine release (adrenalin, noradrenalin) [32]. TNPs also increase HR at rest by 6–10 bpm [22,23]. We previously used higher doses of TNP (Novartis, 20 cm^2^, 35 mg nicotine patch, release rate 14 mg nicotine per 24 h) and reported that the mean HR at rest was significantly higher in the TNP condition than in the control condition (TNP: 63.7 ± 8.2 bpm, control: 56.9 ± 7.3 bpm; *p* = 0.011) [33]. Similarly, HR at rest for the TNP condition in the present study was significantly higher than that for the control condition (TNP; 74.7 ± 13.8 bpm, control; 65.3 ± 10.8 bpm, *p* < 0.001), which is consistent with the findings of previous studies. Furthermore, two-way repeated ANOVA shows no interaction (condition × exercise intensity) in HR responses during exercise between both conditions. However, the HR during exercise was significantly higher in the TNP condition than in the control condition (Figure 1). Furthermore, we examined the relationship between HR and exercise intensity-adjusted covariances with HR at rest, the slope of regression was not significant in both conditions, with the intercept being higher in the nicotine condition (data now shown). This result indicated that TNP did increase HR at baseline, although the relationship between exercise intensity and HR in the TNP condition was almost similar to that in the control condition. Previous studies investigating the effects of nicotine during exercise have reported that nicotine gum and spray, which acutely elevate blood nicotine levels compared to TNPs, increase HR during exercise [24,25,26]. In contrast, a previous study using TNPs during exercise reported that there were no significant differences between TNP and control conditions [34]. Although the administration methods of nicotine are different among gum, spray, and patch applications, the exercise intensity during exercise is suggested as the reason for the differences in HR during exercise. In our study and that of Perkins et al., participants performed exercise at very light to moderate intensity, whereas the participants of Mundel et al.’s study started exercise at a high intensity (77% *V*O2 max) until exhaustion. In general, sympathetic nervous activity and catecholamine levels increase with increasing exercise intensity [20,35]; therefore, the high-intensity exercise and greater sympathetic nervous system activity may have masked the effects of nicotine.

With regard to *V*O_2_, *V*E, and RPE, there were no significant differences between both experiments, which is in accordance with the findings of Mundel et al.’s study [34]. The RPE is a widely used psycho-physical tool to assess subjective perception of effort during exercise [36] and to monitor the intensity of exercise, because it is related to physiological markers of the stress response to exercise [37]. Furthermore, a strong relationship exists between RPE and HR during exercise in general [29]. However, Perkins et al. reported that RPE during exercise at low intensity did not differ between the nicotine spray intake and control conditions [25]. Moreover, in studies using different nicotine concentrations (low to high), HR increased in a nicotine dose-dependent manner (the greater the volume of nicotine, the greater the increase in HR), but it was reported that RPE was not related to nicotine dose [24]. Similarly, there were no significant differences in RPE between our study and Mundel et al.’s study using TNPs. Given that the participants in previous studies, including our study, were healthy young adults, there would be no significant differences in RPE. Although the relationship between nicotine and RPE is unclear in the present study, it is thought that the nicotine products, including TNPs and nicotine spray, do not increase negative bodily sensations, fatigue, and perceived exertion. Further studies are needed to examine the perceived exertion and RPE during exercise with TNPs for other populations.

Our hypothesis was that the La responses during exercise with a TNP and no TNP differ, because nicotine stimulates the sympathetic and parasympathetic nervous systems and secretion of catecholamine (adrenaline and noradrenaline). Previous studies have reported that the catecholamine concentrations during incremental exercise have been shown to start to increase steeply with increasing work intensity, which is similar to the LT [38,39]. However, there were no interactions (condition × exercise intensity) of the main effects of nicotine during exercise between the TNP and control conditions; LT did not differ significantly between both conditions (TNP; 117.8 ± 18.6 W, control; 116.6 ± 17.2 W, *p* = 0.518). As mentioned previously, we applied the TNPs the night before the experiment to achieve the peak nicotine level, and higher HR at rest and during exercise (Figure 1a) is expected when the blood nicotine level is at its peak. However, our results indicate that La concentration at rest and during exercise was not influenced by TNPs.

Smoking cessation reduces the risk of major chronic diseases and extends life expectancy [1,2,3]. In contrast, the relationship between stopping smoking and body weight has been known for many years, with the majority of smokers who attempted to quit smoking gaining body weight, in addition to the nicotine withdrawal symptoms [40,41]. From this background, increasing energy expenditure by exercise per se and/or combined exercise and NRT has received much interest in smokers who desire to stop smoking in the clinical setting. Thus, the implications for exercise prescription during smoking cessation should be discussed here. In the general principles of exercise prescription, exercise intensity is one of the most important areas, as well as frequency, exercise time, and exercise type; direct measurements of HR and *V*O_2_ are recommended for individualized exercise prescription for greater accuracy, but when these are not feasible, estimation of exercise intensity is acceptable [37]. In general, HR monitoring using an electrocardiogram (ECG) monitor or automated HR monitor during exercise and exercise prescription based on HR and RPE have become an established and common method in the clinical setting. However, assuming exercise prescriptions at the time of smoking cessation using TNPs, given that TNPs increases HR at rest, the HR reserve (HRR) methods may be preferable for exercise prescription as compared to the %HRmax method. If the exercise program used the %HR method despite the same value of RPE, exercise intensity could be underestimated as compared to the HRR method. As an alternative method of setting the exercise intensity, a method measuring LT is useful. Performing at LT is safe and favorable on aerobic capacity and metabolic syndrome and related disorders, such as hypertension and glucose intolerance [42,43,44].

However, our study has several limitations. First, all the study participants were healthy young men. The smoking history of the participants in this study was shorter by several years than those of previous studies, but the cardiorespiratory responses during exercise with TNP in other populations might be different from those in our younger participants. For example, Fletcher and Peto [45] reported that the reduction in forced expiratory volume in one second (FEV1) for smokers became apparent after 40 years of age, as compared to that of non-smokers. Moreover, previous studies have shown that women metabolize nicotine more rapidly than men [46,47]. It is unclear whether our findings on the influence of smoking history and sex on exercise tolerance can be applied to other populations. Second, we did not measure the BP level at rest and during exercise, and further studies are still needed. Previous studies reported that nicotine increases both HR and BP levels in smokers [22,23], and we speculated that the BP level was also higher in the TNP condition in our young men. Furthermore, the double product (HR × systolic BP), which is considered to be a useful index of myocardial oxygen uptake [38] during exercise would be higher in the TNP condition. Third, we used a lower dose of TNPs in this study, although generally smokers may try a higher dose of TNPs at the beginning of smoking cessation. Therefore, the cardiorespiratory responses, particularly HR, will increase in higher doses of TNPs. Fourth, the present study used aerobic-type exercises. Recently, it was found that anaerobic-type exercises, such as resistance exercises, also have the potential to reduce nicotine withdrawal symptoms [48,49]. The exercise type and energy profile of these exercises are different than in aerobic-type exercises at light to moderate intensity, whereas resistance exercise using machines or free-weights at high intensity acutely increases HR and blood pressure up to 140–160 bpm (80% HRmax) [50,51]. Therefore, physiological responses during exercise combined with TNPs could differ between aerobic-type exercises and anaerobic-type exercises, and a further study is needed to examine cardiorespiratory responses during other types of exercises. Last, the present study is a random crossover single-blind design. Because double-blind design is more reliable than single-blind, further studies with a double-blind design are needed to examine the effect of TNPs.

## 5. Conclusions

When comparing the cardiorespiratory responses and La concentration during exercise at light to moderate intensity between TNP and control conditions, the HR during exercise was 6.7 bpm higher on average in the TNP condition than in the control condition. However, there were no significant differences in *V*O_2_, *V*E, RPE, and La between both conditions. For practical recommendations for exercise prescription during smoking cessation in the clinical setting, clinicians should consider that TNPs increase the HR at rest and during exercise when prescribing exercise intensity and monitoring physiological responses, and the HRR methods may be preferred when prescribing the exercise intensity based on HR. The results of our study are valuable for clinicians and health professionals to guide them when prescribing exercise programs combined with TNPs during smoking cessation in the clinical setting, such as cardiology and respiratory medicine, for healthy young individuals.

## Figures and Tables

**Table 1:** Participants’ characteristics (mean ± SD, 95% confidence interval).

Variables	Age (years)	Height (cm)	Weight (kg)	BMI (kg/m^2^)
Smokers, *n* = 6	21.7 ± 1.4 * (20.2, 23.1)	177.8 ± 4.6 * (172.7, 182.3)	72.3 ± 15.7 (56.0, 88.8)	22.7 ± 4.0 (18.5, 26.9)
Non-smokers, *n* = 8	24.2 ± 2.3 (22.6, 25.8)	172.2 ± 5.2 (168.7, 176.0)	64.8 ± 5.4 (61.0, 68.7)	21.8 ± 1.1 (21.0, 22.6)
Total, *n* = 14	23.3 ± 2.3 (22.0, 24.5)	174.3 ± 5.6 (171.4, 177.1)	67.6 ± 10.6 (62.0, 73.3)	22.2 ± 2.5 (20.8, 23.5)

Note: SD, standard deviation; BMI, body mass index; *Significantly different vs. non-smokers, *p* < 0.05.
